# Biomass Estimation of Dry Tropical Woody Species at Juvenile Stage

**DOI:** 10.1100/2012/790219

**Published:** 2012-02-15

**Authors:** R. K. Chaturvedi, A. S. Raghubanshi, J. S. Singh

**Affiliations:** ^1^Ecosystems Analysis Laboratory, Department of Botany, Banaras Hindu University, Varanasi 221005, India; ^2^Institute of Environment and Sustainable Development, Banaras Hindu University, Varanasi 221005, India

## Abstract

Accurate characterization of biomass in different forest components is important to estimate their contribution to total carbon stock. Due to lack of allometric equations for biomass estimation of woody species at juvenile stage, the carbon stored in this forest component is ignored. We harvested 47 woody species at juvenile stage in a dry tropical forest and developed regression models for the estimation of above-ground biomass (AGB). The models including wood-specific gravity (*ρ*) exhibited higher *R* ^2^ than those without *ρ*. The model consisting of *ρ*, stem diameter (*D*), and height (*H*) not only exhibited the highest *R* ^2^ value but also had the lowest standard error of estimate. We suggest that *ρ*-based regression model is a viable option for nondestructive estimation of biomass of forest trees at juvenile stage.

## 1. Introduction

For accurate estimation of carbon sink in the forest, a precise mapping of forest biomass at a fine resolution is required [1]. Generally, forest biomass is estimated by a common allometric equation which is generally applied over a large area [2]. Variety of factors such as age of stand, species, topography, environmental heterogeneity, and human disturbance, however, affect forest biomass. Therefore, a considerable uncertainty exists in the estimation of spatial distribution of biomass [3, 4]. Several authors [5–12] have published biomass estimations using species-specific allometric equations relating destructively measured tree biomass and field measured circumference at breast height (CBH) or diameter at breast height (DBH) for trees with CBH >10 cm. Recently, Singh et al. [13] have established allometric equations for both above- and below-ground components of three native species (diameter <10 cm) used for plantation in the Indian subcontinent. Most biomass equations have only CBH or DBH as estimator, causing a significant problem for regional-scale comparisons of tree biomass estimates. In order to explore the variations in biomass estimates due to environmental, structural, and compositional gradients, wood-specific gravity (*ρ*) has been incorporated as a simple multiplication factor in diameter-based biomass equations based on tree diameter (e.g., [14–18]).

Individuals in small stem circumference class (<10 cm) comprise a significant proportion of tree population and have faster growth rate than the higher diameter class trees, but allometric equations for their biomass estimation are lacking. As a result, the carbon stored in the juvenile tree population is ignored. In this study, we measured above-ground biomass (AGB) of 47 woody species at juvenile stage, occurring in dry tropical forest, by harvest method and developed a multispecies regression model for the nondestructive estimation of AGB with the help of wood-specific gravity (*ρ*), stem diameter (*D*), and plant height (*H*). Further, we observed the strength of similarity between the species-specific, actual harvested AGB, and the AGB estimated by the multispecies regression model.

## 2. Materials and Methods

We harvested 10 juvenile individuals of each of the 47 woody species in the dry deciduous forest (21°29′–25°11′ N lat. and 78°15′–84°15′ E long.) of Vindhyan highlands situated in Sonebhadra District of Uttar Pradesh, India. The juvenile individuals represented the population of each species in the forest at juvenile stage. The juvenile stage was defined as individuals having ≥30 cm height and <10 cm stem circumference 10 cm above the ground surface. Height (*H*) and stem circumference of each individual were recorded. Leaves were plucked and stem and branches were cut into small pieces. From each individual plant, wood samples were taken and *ρ* was estimated following the method described by Chaturvedi et al. [14]. For the estimation of above-ground dry biomass (AGB), stem, branches, and leaves of each individual plant were dried in an oven at 80°C to constant weight. Data of all the species were pooled to develop regression models for the estimation of AGB on the basis of *D*, *H*, and *ρ*. For *Dendrocalamus strictus*, a separate model was developed. The best model was selected on the basis of *R* ^2^ and standard error of estimate. To check for the strength of relationship between AGB estimated for individual species by harvest method and that by the multispecies regression model, we regressed the harvest data of 12 dominant species against the estimates obtained through the newly developed model.

## 3. Results and Discussion

Ranges of *ρ*, *D*, and *H* are shown in Table 1. The values of measured *ρ* reported in this study for juvenile trees are lower compared to those reported for mature trees of dry tropical forest [19, 20] and were higher for individuals with greater *D*. The regression models developed for the estimation of AGB are reported in Table 2. All these models explained more than 80% variability in AGB. The models including *ρ* exhibited higher *R* ^2^ than those without *ρ*. The model consisting of *ρ*, *D*, and *H* not only exhibited the highest *R* ^2^ value but also had the lowest standard error of estimate.

**Figure 1:**
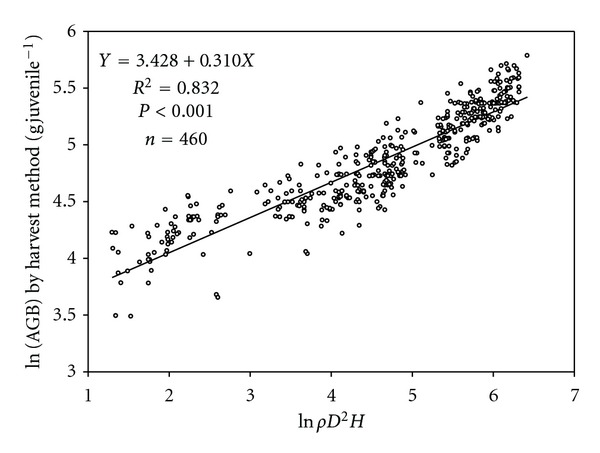
Relationships between the log transformed values of *ρD* ^2^ *H* and the log transformed values of above-ground biomass (AGB, g) estimated by harvest method for 46 juvenile tree species. *ρ*: wood-specific gravity ( g cm^−3^); *D*: stem diameter (cm); *H*: height (cm).

**Figure 2:**
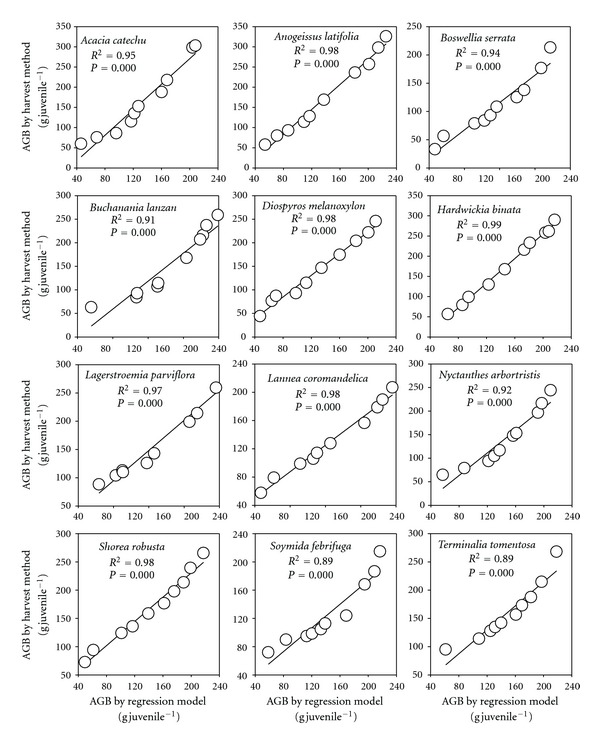
Relationships between the above ground biomass (AGB) of the dominant juvenile tree species estimated by allometric equation and by harvest method.

We selected model 4 (see Table 2) as the most suitable model for the estimation of AGB of the juvenile woody species.  Figure 1 shows strong correlation between harvested AGB and the estimator used in our model. Average AGB for all species excluding *Dendrocalamus strictus* estimated by harvest method was 138 ± 2.26 g juvelile^−1^ and that from regression model was 141 ± 2.83 g juvelile^−1^. The model was also validated against harvested AGB of 12 dominant species of the forest (Figure 2). In *Dendrocalamus strictus*, the *H* of plant was greater at a particular *D* as compared to other species, making its value an outlier in the regression analysis. Therefore, we developed a separate model for *Dendrocalamus strictus* as *Y* = 2.487 + 0.414*X*, *R* ^2^ = 0.967, and *P* < 0.001, where *Y* = ln⁡⁡AGB and *X* = ln⁡*ρD* ^2^ *H*. For this species, the average AGB estimated by harvest method was 110 ± 16.6 g juvelile^−1^ and that from regression model was 112 ± 19.1 g juvelile^−1^. 

Chave et al. [21] also reported *ρ* as an important predictive variable in different regression models developed for the estimation of AGB in tropical forests. Since the density of carbon per unit volume is highly correlated with *ρ*, it has direct implication for estimating ecosystem carbon storage and fluxes [15, 16, 22, 23]. Muller-Landau [24] found significant difference in *ρ* of tropical trees among sites. For the estimation of sitewise variation of biomass of a woody species at juvenile stage, a common site-specific *ρ* value was obtained by harvesting few individuals and can be applied in the regression model for all the individuals of the species at a site. Thus, the regression model can become a viable option for nondestructive estimation of biomass of forest tree component at juvenile stage.

Most of the current studies of biomass estimation are focused on relatively large trees (>10 cm diameter) ignoring the contribution of other forest components particularly juvenile woody species. In some situations, these types of studies could be justified; however, in forests such as dry tropical forests which are continuously under high anthropogenic disturbance, the biomass estimation of low diameter trees becomes particularly important. Sagar and Singh [25] have reported 85% of individuals in the dry tropical forest being at juvenile stage at any given time. Therefore, to understand the carbon dynamics, it is necessary to include juveniles in biomass estimation programmes.

## Figures and Tables

**Table 1:** Range of stem diameter (*D*), height (*H*), and wood-specific gravity (*ρ*) of the juvenile tree species harvested from the study site. *n* = 10 per species.

S. no.	Species	*ρ* (g cm^−3^)	*D* (cm)	*H* (cm)
(1)	*Acacia auriculiformis*	0.47–0.52	0.5–3.1	30.5–103.4
(2)	*Acacia catechu*	0.48–0.58	0.6–2.9	30.8–99.2
(3)	*Adina cordifolia*	0.34–0.38	0.6–3.1	31.4–112.6
(4)	*Albizia odoratissima*	0.47–0.57	0.7–2.7	30.7–94.5
(5)	*Anogeissus latifolia*	0.56–0.64	0.6–3.1	30.6–108.8
(6)	*Azadirachta indica*	0.52–0.58	0.5–2.9	31.6–98.3
(7)	*Bauhinia racemosa*	0.52–0.58	0.9–3.1	31.5–99.5
(8)	*Boswellia serrata*	0.34–0.38	0.6–2.8	30.4–98.0
(9)	*Bridelia retusa*	0.48–0.56	0.8–3.0	30.7–98.6
(10)	*Buchanania lanzan*	0.45–0.56	0.7–2.9	31.7–88.9
(11)	*Carissa spinarum*	0.52–0.57	0.6–3.1	30.6–89.7
(12)	*Cassia fistula*	0.51–0.56	0.8–2.6	30.4–91.0
(13)	*Cassia siamea*	0.53–0.59	0.6–3.1	30.5–111.7
(14)	*Chloroxylon swietenia*	0.47–0.54	0.7–3.1	31.6–99.6
(15)	*Dendrocalamus strictus*	0.45–0.49	0.7–2.8	60.8–196.0
(16)	*Diospyros melanoxylon*	0.53–0.58	0.5–3.1	30.8–89.6
(17)	*Elaeodendron glaucum*	0.51–0.57	0.7–3.0	30.5–99.4
(18)	*Emblica officinalis*	0.53–0.58	0.6–2.8	30.6–117.0
(19)	*Flacourtia indica*	0.55–0.59	0.7–3.1	31.4–99.7
(20)	*Gardenia latifolia*	0.48–0.53	0.8–2.9	30.5–98.3
(21)	*Gardenia turgida*	0.51–0.55	0.7–2.8	31.6–98.0
(22)	*Grewia hirsuta*	0.48–0.53	0.6–3.1	30.6–99.4
(23)	*Grewia serrulata*	0.51–0.55	0.8–2.8	30.7–98.0
(24)	*Hardwickia binata*	0.58–0.65	0.8–2.9	30.4–98.7
(25)	*Holarrhena antidysenterica*	0.52–0.55	0.6–2.8	30.8–98.0
(26)	*Holoptelea integrifolia*	0.52–0.58	0.5–3.1	31.4–99.4
(27)	*Hymenodictyon excelsum*	0.48–0.54	0.8–3.0	30.8–99.5
(28)	*Indigofera cassioides*	0.48–0.52	0.7–2.9	30.3–98.3
(29)	*Lagerstroemia parviflora*	0.52–0.57	0.8–2.8	31.2–98.0
(30)	*Lannea coromandelica*	0.35–0.41	0.6–3.1	30.5–116.3
(31)	*Lantana camara*	0.42–0.46	0.8–2.8	30.4–98.0
(32)	*Madhuca longifolia*	0.47–0.54	0.7–2.9	30.8–98.5
(33)	*Miliusa tomentosa*	0.52–0.56	0.6–3.1	31.0–99.7
(34)	*Mitragyna parvifolia*	0.51–0.59	0.7–2.9	30.4–98.6
(35)	*Nyctanthes arbortristis*	0.48–0.53	0.7–3.1	31.4–99.3
(36	*Ougeinia oogenesis*	0.51–0.54	0.6–3.1	30.7–99.2
(37)	*Pterocarpus marsupium*	0.58–0.67	0.6–2.7	30.6–94.5
(38)	*Schleichera oleosa*	0.51–0.54	0.5–3.1	30.8–99.6
(39)	*Schrebera swietenioides*	0.51–0.58	0.6–2.9	31.6–98.4
(40)	*Semecarpus anacardium*	0.41–0.46	0.6–3.1	30.5–99.4
(41)	*Shorea robusta*	0.61–0.67	0.5–2.9	30.7–119.6
(42)	*Soymida febrifuga*	0.53–0.58	0.7–3.1	31.7–98.8
(43)	*Terminalia tomentosa*	0.61–0.67	0.7–2.9	30.7–98.5
(44)	*Woodfordia fruticosa*	0.49–0.55	0.8–2.9	30.6–87.9
(45)	*Zizyphus glaberrima*	0.48–0.55	0.7–3.0	31.4–98.5
(46)	*Zizyphus nummularia*	0.52–0.56	0.9–2.9	31.5–98.7
(47)	*Zizyphus oenoplea*	0.47–0.53	0.6–3.1	30.4–89.4
	Average	0.53	1.90	67.8

**Table 2:** Regression models for estimating biomass of juvenile trees. SEE: Standard error of estimate. *Y*: logarithm of above ground biomass (ln⁡AGB); *X*_1_ : *lnD* ^2^; *X*_2_ : *l* *n* *ρD* ^2^; *X*_3_ : *lnD* ^2^ *H*; *X*_4_ : *l* *n* *ρD* ^2^ *H*.

S. no.	Model	*R* ^2^	SEE	*P*
(1)	*Y* = 3.344 + 0.443*X*_1_	0.809	0.196	< 0.001
(2)	*Y* = 2.666 + 0.432*X*_2_	0.824	0.188	< 0.001
(3)	*Y* = 3.204 + 0.315*X*_3_	0.819	0.191	< 0.001
(4)	*Y* = 3.428 + 0.310*X*_4_	0.832	0.184	< 0.001
